# Trajectories of self-care and task execution abilities among older patients with chronic heart failure following a nurse-led intervention: A longitudinal mixed-effects analysis

**DOI:** 10.1016/j.ijnsa.2026.100619

**Published:** 2026-07-14

**Authors:** Haixiang Zhu, Yan Li, Xiaoxue Han, Jinxuan Chen, Ruiting Wang, Xiuruo Zhu

**Affiliations:** a Nursing Department, Sir Run Run Shaw Hospital, Zhejiang University School of Medicine, China; b Wenzhou People's Hospital, Wenzhou, Zhejiang Province, China

**Keywords:** Chronic heart failure, Self-care ability, Task execution ability, Nurse-led intervention

## Abstract

**Aim and objectives:**

To examine longitudinal changes in self-care ability and task execution ability among older patients with chronic heart failure following a nurse-led age-friendly graded symptom-recognition intervention.

**Background:**

Older patients with chronic heart failure often experience difficulties in symptom recognition, response selection, and sustained self-management after discharge. Although self-care has been widely emphasized in heart failure management, less is known about how nurse-led interventions influence the development of both self-care ability and task execution ability over time, particularly during the vulnerable post-discharge transition period.

**Design:**

This study was a secondary analysis based on a previously published randomized controlled trial protocol in older patients with chronic heart failure recruited from a tertiary hospital. Participants were originally assigned to either a nurse-led age-friendly graded symptom-recognition intervention plus usual care or usual care alone. The present analysis focused on behavioral outcomes within the first 3 months after discharge.

**Methods:**

Self-care ability and task execution ability were assessed at baseline, 1 month, and 3 months after discharge. Self-care ability was measured using the Chinese version of the European Heart Failure Self-care Behaviour Scale (EHFScBS-9), and task execution ability was evaluated using a structured task performance scale assessing key self-management tasks. Longitudinal trajectories were analysed using linear mixed-effects models.

**Results:**

For self-care ability, significant main effects of group and time were observed, along with a significant group × time interaction (F_group = 11.65, P < 0.001; F_time = 5.74, P = 0.003; F_interaction = 8.10, P < 0.001). The standardized interaction effect increased from modest at 1 month (β ≈ 0.30) to large at 3 months (β ≈ 1.00). For task execution ability, no significant group main effect was observed (F_group = 0.01, P = 0.91), whereas significant time and interaction effects were detected (F_time = 36.19, P < 0.001; F_interaction = 26.91, P < 0.001). The interaction effect was moderate at 1 month (β ≈ 0.65) and large at 3 months (β ≈ 1.35).

**Conclusions:**

A nurse-led age-friendly graded symptom-recognition intervention was associated with improved longitudinal patterns of both self-care ability and task execution ability among older patients with chronic heart failure. These findings suggest that structured symptom-recognition support may strengthen patients’ ability to translate symptom cues into appropriate self-management actions during the vulnerable post-discharge transition period.

**What is already known**

• Older patients with chronic heart failure often experience difficulties in symptom recognition, response selection, and sustained self-management after discharge.
• Self-care is essential in heart failure management, but self-reported measures may not fully capture patients’ ability to perform concrete self-management tasks.
• Evidence on how nurse-led interventions influence longitudinal changes in both self-care ability and task execution ability remains limited.

Alt-text: Unlabelled box dummy alt text

**What this paper adds**

• A nurse-led age-friendly graded symptom-recognition intervention was associated with improved longitudinal patterns of both self-care ability and task execution ability in older patients with chronic heart failure.
• Task execution ability showed earlier and more sustained divergence between groups than self-care ability, suggesting that it may be a sensitive behavioral target during the post-discharge transition period.
• Symptom monitoring-related domains appeared particularly responsive to structured reinforcement, highlighting the importance of targeted post-discharge support.

Alt-text: Unlabelled box dummy alt text

## 1 Introduction

Chronic heart failure (CHF) is a progressive clinical syndrome associated with high prevalence, frequent hospitalization, and substantial mortality worldwide, placing a considerable burden on patients, families, and healthcare systems (Huang et al., 2022). Despite advances in pharmacological and device-based therapies, long-term outcomes remain suboptimal, largely because of recurrent decompensation and preventable readmissions. Effective self-care is therefore recognized as a cornerstone of CHF management, particularly in older patients, who are more vulnerable to functional decline, recurrent exacerbation, and adverse prognosis (Yu et al., 2022).

Self-care in CHF involves a range of daily health-related behaviors, including symptom monitoring, medication adherence, dietary and fluid management, and timely responses to clinical deterioration. Inadequate self-care has been associated with poorer quality of life, higher rehospitalization risk, and increased mortality in patients with CHF (Dorsch et al., 2025; Wang et al., 2025). However, self-care is commonly assessed using self-reported measures, which may not fully reflect whether patients can independently perform essential disease-management tasks in real-world settings (Calero-Molina et al., 2022; Vidán et al., 2019). Task execution ability refers to the capacity to correctly and autonomously carry out core self-management tasks, such as monitoring body weight, identifying edema or high-sodium foods, and managing medications, without external prompting (Vidán et al., 2019). In older patients with CHF, this ability is closely linked to symptom-oriented self-management, because effective disease control depends not only on perceiving symptom changes but also on translating these changes into timely and appropriate actions. Emerging evidence suggests that task execution ability represents a fundamental and objective component of self-care and may be more closely associated with disease severity and adverse outcomes than self-reported behaviors alone. Prior cross-sectional studies, including our own, have shown that task execution ability in older patients with CHF is generally poor and is significantly associated with cardiac functional status, biomarker profiles, and mortality-related risk indicators (Zhu et al., 2025). These findings identify task execution ability as an important but insufficiently addressed intervention target. Conceptually, task execution ability is related to, but distinct from, self-care ability. Self-care ability reflects a broader capacity to maintain health-related behaviors and respond to symptom deterioration, whereas task execution ability represents the more proximal capacity to carry out concrete self-management tasks in daily life. Within heart failure self-care, symptom recognition and response selection can be regarded as upstream cognitive processes, while task execution reflects the behavioral enactment through which these cognitive processes are translated into observable self-management actions.

This issue is especially salient in older patients with CHF. Symptom deterioration is often gradual, nonspecific, and difficult to interpret, particularly among older adults with reduced functional reserve, sensory decline, and limited capacity to integrate symptom cues with complex self-care instructions. As a result, patients may recognize that their condition has changed but still be unable to judge symptom severity, select an appropriate response, or seek care in a timely manner. This gap between symptom perception and behavioral enactment may contribute directly to delayed healthcare seeking and poorer outcomes. In this context, task execution ability may be understood as the operational bridge between symptom recognition and real-world self-management behavior. When this bridge is weak, adequate disease knowledge alone may be insufficient to produce effective action. Improving this operational bridge remains challenging in older patients with CHF, because cognitive decline, reduced functional reserve, and limited capacity to transform disease knowledge into sustained action may weaken the continuity between symptom appraisal and behavioral enactment. Existing self-management interventions have largely focused on education or short-term behavior change, and less is known about whether such interventions can produce sustained improvements in concrete task execution over time.

Nurse-led interventions are well positioned to address these challenges. Nurses play a central role in CHF management through education, skills training, ongoing assessment, and behavioral reinforcement. Age-friendly implementation aids may be particularly useful for older patients because they simplify complex symptom information into structured, visually supported, and action-oriented guidance. Such approaches may reduce cognitive burden, improve recognition of worsening symptoms, and strengthen the link between symptom perception and appropriate response. Structured nurse-led programs that integrate symptom recognition, decision support, and practical skill training may therefore promote not only better self-care but also more durable gains in task execution ability (Riegel et al., 2022). By improving the pathway from symptom identification to action selection, age-friendly graded symptom-recognition interventions may also reduce delayed or inappropriate responses to symptom worsening and thereby improve behavioral management during the vulnerable post-discharge transition period.

The present study was conceptually guided by a heart failure self-care framework (Huang et al., 2022; Riegel et al., 2022) and a task-oriented interpretation of self-management. In this perspective, effective CHF self-care involves interrelated processes of symptom perception, self-care management, and self-care maintenance. For older patients, these processes may be disrupted by symptom ambiguity, reduced cognitive reserve, limited confidence in symptom appraisal, and difficulty transforming instructions into action. The nurse-led age-friendly graded symptom-recognition intervention was therefore conceptualized as acting first on symptom perception and response selection by making symptom cues more visible, risk levels more explicit, and recommended actions more concrete. Through repeated demonstration, scenario-based practice, reinforcement, and feedback, the intervention may further strengthen task execution ability, namely the ability to perform specific self-management tasks such as monitoring, recording, assessing oedema, identifying high-salt foods or prescribed diuretics, and taking appropriate action. Improved task execution ability may then support more stable self-care ability over time and may plausibly contribute to downstream clinical outcomes, such as timely care-seeking and reduced rehospitalization. This proposed conceptual pathway is summarized in Supplementary Figure S1. Despite this theoretical plausibility, few studies have empirically examined whether nurse-led symptom-recognition interventions are associated with longitudinal changes in both self-care ability and task execution ability among older patients with CHF. In particular, limited evidence is available on whether improvements in task execution ability emerge earlier than changes in broader self-care ability, or whether specific domains of task execution are more responsive to structured reinforcement. Therefore, the present study aimed to examine the longitudinal changes in self-care ability and task execution ability among older patients with CHF during the vulnerable post-discharge transition period following a nurse-led age-friendly graded symptom-recognition intervention. Using mixed-effects modeling, this study sought to characterize time-dependent changes and group-by-time interactions in overall and domain-specific outcomes to clarify how these core self-management capacities evolved over time.

## 2 Methods

### 2.1 Study design and participants

**Fig. 1:**
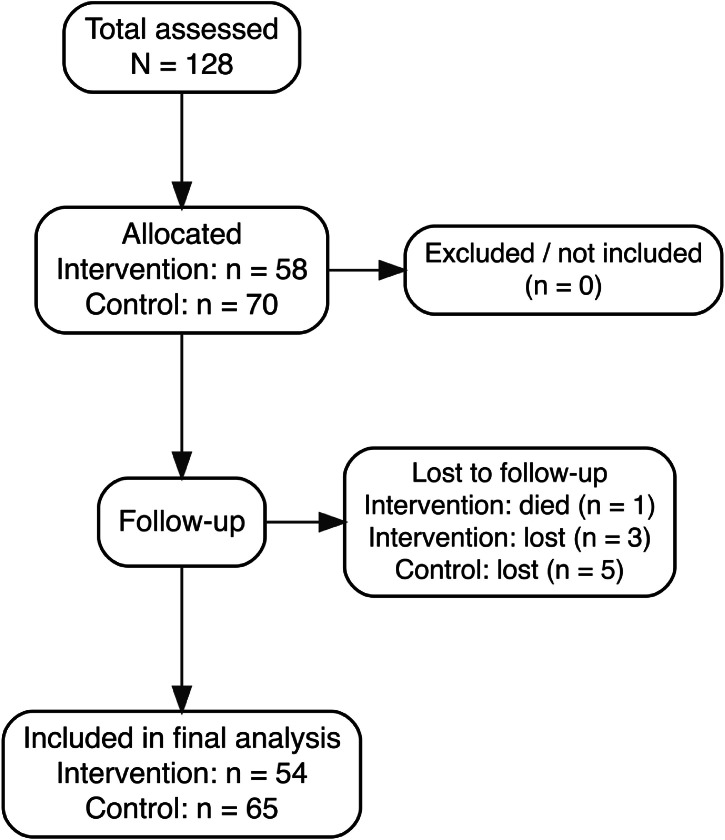
Participant flow diagram.

This study was a secondary analysis derived from a previously published randomized controlled trial protocol evaluating a nurse-led graded symptom-recognition intervention for older patients with chronic heart failure (CHF) (Zhu et al., 2026). The parent trial was designed to examine clinical outcomes such as delayed healthcare-seeking behavior and healthcare utilization, whereas the present analysis focused on behavioral outcomes, specifically self-care ability and task execution ability, to explore the short-term behavioral mechanisms of the intervention. The parent trial was conducted in a tertiary hospital in Zhejiang Province, China. In the present analysis, behavioral outcomes were assessed at baseline, 1 month after discharge, and 3 months after discharge. The current study focused on the vulnerable post-discharge transition period, which is critical for the consolidation of self-management behaviors in older patients with CHF. The study protocol was approved by the institutional ethics committee (Approval No. 2025-Y0513), and all procedures were conducted in accordance with the principles of the Declaration of Helsinki. All participants received verbal and written information about the study and provided written informed consent before participation. For the parent trial, sample size estimation was based on delayed healthcare-seeking behavior as the primary outcome. After accounting for an anticipated attrition rate of approximately 10%, the target sample size was 148 participants (74 per group), as described in the published protocol. During the study period, 128 patients were allocated in the parent trial, and 119 participants with complete behavioral outcome data at the required follow-up points were included in the present longitudinal analysis (65 in the control group and 54 in the intervention group), as shown in Fig. 1. Because the present analysis focused on behavioral outcomes, participants with incomplete follow-up data for self-care ability or task execution ability were excluded to ensure analytical reliability. To be eligible for participation, individuals had to meet the following criteria: (a) a diagnosis of CHF according to current guideline criteria (Calero-Molina et al., 2022); (b) age ≥60 years; (c) sufficient cognitive ability to complete study assessments; and (d) clinical stability at discharge. Patients were excluded if they had severe comorbid conditions that limited participation or had taken part in similar intervention programs within the preceding month.

A total of 128 patients with chronic heart failure were assessed and allocated to the intervention group (n = 58) or control group (n = 70). During follow-up, one participant in the intervention group died and three were lost to follow-up, while five participants in the control group were lost to follow-up. Ultimately, 54 participants in the intervention group and 65 in the control group were included in the final analysis.

### 2.2 Randomization and allocation concealment

Participants were randomly assigned in a 1:1 ratio to either the intervention group or the control group after written informed consent and completion of baseline assessment. The random allocation sequence was generated using a computer-generated randomization list by an independent researcher who was not involved in recruitment, intervention delivery, outcome assessment, or data analysis. To ensure allocation concealment, group assignments were placed in sequentially numbered, opaque, sealed envelopes. The envelopes were prepared and kept by an independent researcher who was not involved in recruitment, intervention delivery, outcome assessment, or data analysis. After baseline assessment, the next envelope in numerical order was opened to determine group assignment. The allocation result was communicated only to the nurses responsible for intervention delivery. Outcome assessors and data analysts remained blinded to group allocation throughout data collection and analysis.

### 2.3 Intervention

#### 2.3.1 Usual care

Both groups received usual care during hospitalization and routine follow-up after discharge. Usual care included standard verbal and written education on heart failure, medication adherence, dietary management, physical activity, and general symptom monitoring, together with routine post-discharge follow-up according to ward practice. Patients in the control group received routine discharge education and scheduled follow-up, but did not use the age-friendly implementation aids.

#### 2.3.2 Nurse-led age-friendly implementation program

In addition to usual care, patients in the intervention group received a nurse-led age-friendly implementation program based on a graded symptom-recognition approach (Zhu et al., 2026), the age-friendly graded symptom-recognition tool is provided as supplementary File 1. Detailed components and procedures of the intervention are presented in Table 1. In the parent trial, the intervention consisted of several age-friendly implementation aids designed to support early symptom recognition and appropriate response behaviors, including a graded symptom-recognition tool, a structured recording aid, and a supportive edema assessment component. These tools were intended to help patients identify symptom severity and select corresponding response strategies, thereby establishing a structured perception–decision–action pathway.

**Table 1:** Nurse-led age-friendly graded symptom-recognition intervention program.

Time	Theme	Goal	Implementer	Content and methods
Within 24 h of admission	Initial one-to-one training	To establish patients’ and caregivers’ basic understanding of the intervention and of symptom recognition and response.	Trained registered nurses	One-to-one education sessions lasting 20–30 min were delivered to patients and their primary caregivers. The sessions introduced the age-friendly implementation aids, including the graded symptom-recognition tool, the structured recording aid, and the edema assessment aid. Education covered the purpose of the tools, symptom grading principles, response strategies, and the patient’s role in daily self-management. Demonstration, explanation, and immediate feedback were provided.
During hospitalization	Symptom-recognition tool training	To help patients correctly identify common worsening CHF symptoms and match symptom severity with corresponding actions.	Trained registered nurses	The graded symptom-recognition tool was used to teach recognition of core deterioration-related symptoms, particularly dyspnea, edema, and weight change. Symptoms were categorized into graded risk levels, each linked to predefined action strategies. Teaching was delivered using age-friendly visual cues and scenario-based demonstration.
During hospitalization	Use of structured recording aid	To improve consistency of daily symptom monitoring and standardize self-recording.	Trained registered nurses	Patients were instructed to use a simplified structured recording aid with preprinted symptom items and check boxes to document daily symptom changes, self-assessment results, and actions taken. Nurses demonstrated how and when to complete the recording form and reinforced regular documentation habits.
During hospitalization	Edema assessment training	To improve the accuracy of peripheral edema recognition and reduce subjective judgment errors.	Trained registered nurses	The edema assessment aid was demonstrated and practiced with patients and caregivers to support standardized observation of limb swelling. Nurses explained how to compare current findings with baseline status and how to integrate edema changes into the graded symptom-recognition process.
Day 2, day 4, and discharge day	Reinforcement and competency assessment	To ensure correct understanding, accurate operation, and alignment between patient self-assessment and nurse evaluation before discharge.	Trained registered nurses	Reinforcement assessments were conducted using a structured assessment form. Assessment focused on tool understanding, operational accuracy, recording consistency, and concordance between patients’ self-assessments and nurses’ evaluations. Targeted re-education was provided when necessary. Patients were required to demonstrate correct understanding and operation of key items before completing the in-hospital intervention phase.
Before discharge	Predischarge review and transition preparation	To prepare patients for independent use of the intervention aids after discharge and strengthen symptom-triggered action readiness.	Trained registered nurses	Nurses reviewed the use of the graded symptom-recognition tool, the recording aid, and the edema assessment aid; checked patients’ understanding of symptom-triggered responses; and clarified when to continue observation, adjust management, or seek medical care. Patients were instructed to continue daily self-assessment and recording after discharge.
After discharge (daily)	Daily self-assessment and self-recording	To promote continuous symptom monitoring, early detection of deterioration, and timely response.	Patient / primary caregiver	Patients were instructed to perform daily self-assessment using the graded symptom-recognition framework and record results using the structured recording aid. Symptom changes triggered immediate reassessment. Moderate- or high-risk findings required prompt submission or communication for clinical review according to protocol.
First 4 weeks after discharge	Standardized telephone follow-up	To reinforce adherence, improve accuracy of tool use, and provide individualized re-education during the early high-risk postdischarge period.	Trained registered nurses not involved in outcome assessment	Telephone follow-up was scheduled once weekly during the first 4 weeks after discharge. Follow-up included assessment of adherence and accuracy, clarification of symptom interpretation, targeted re-education, and documentation of all interactions.
From week 5 onward (frequency adjusted by performance)	Tailored follow-up intensity	To provide dynamic support according to patients’ actual performance and prevent decline in adherence or accuracy.	Trained registered nurses not involved in outcome assessment	Patients with adherence below 80% or accuracy below 90% received more intensive follow-up (2–3 times per week). Patients with high adherence (≥95% for 4 consecutive weeks) were followed once monthly starting from week 5.
Throughout the intervention period	Intervention fidelity monitoring	To ensure standardized delivery and reduce implementation variability.	Principal investigator and trained nurses	All nurses received standardized protocol-based training before implementation. Standardized materials, structured assessment forms, predefined follow-up procedures, and documentation requirements were used. Intervention adherence, accuracy, and delivery records were monitored throughout the study period.

Note: CHF, chronic heart failure.

**In-hospital training**:In-hospital training was initiated within 24 hours of admission. Trained research nurses conducted one-to-one education sessions with patients and their primary caregivers, lasting 20–30 minutes. The sessions covered the purpose of the intervention aids, item-by-item explanation of the graded symptom-recognition tool, practical demonstration of the edema assessment component, guidance on structured recording, and simulated scenario practice with immediate feedback.

**In-hospital reinforcement and discharge competency assessment:** During hospitalization, reinforcement assessments were conducted on the second day, fourth day, and the day of discharge by trained research nurses using a structured assessment form. These assessments focused on tool understanding, operational accuracy, recording consistency, and concordance between patients’ self-assessments and nurses’ evaluations. Targeted reinforcement was provided when necessary, and patients were required to demonstrate correct understanding and operation of key items before completing the in-hospital intervention phase.

**Post-discharge self-management and follow-up:**After discharge, patients in the intervention group were instructed to perform daily self-assessment and recording using the intervention aids. Symptom changes triggered immediate self-assessment and submission for clinical review when required.

Standardized follow-up was conducted by trained registered nurses who were not involved in outcome assessment. Telephone follow-up was scheduled once weekly during the first 4 weeks after discharge. Patients with adherence below 80% or accuracy below 90% received more intensive follow-up, at a frequency of 2–3 times per week. Patients who maintained high adherence (≥95% for 4 consecutive weeks) were followed once monthly starting from week 5 onward. Follow-up included assessment of adherence and accuracy, targeted re-education, and full documentation of all interactions.

**Intervention fidelity:**To ensure standardized implementation, all registered nurses involved in intervention delivery completed protocol-based training before study initiation. Standardized educational scripts, structured assessment forms, and predefined follow-up procedures were used throughout the study. Nurses responsible for intervention delivery and follow-up were not involved in outcome assessment. Adherence to the intervention process, including in-hospital training, reinforcement assessments, post-discharge follow-up, and documentation, was monitored throughout the study period.

Although the parent trial included a 6-month intervention period, the present analysis focused on behavioral outcomes within the first 3 months after discharge. The overall components and delivery procedures of the intervention are summarized in Table 1.

### 2.4 Data collection and quality control

**Fig. 2:**
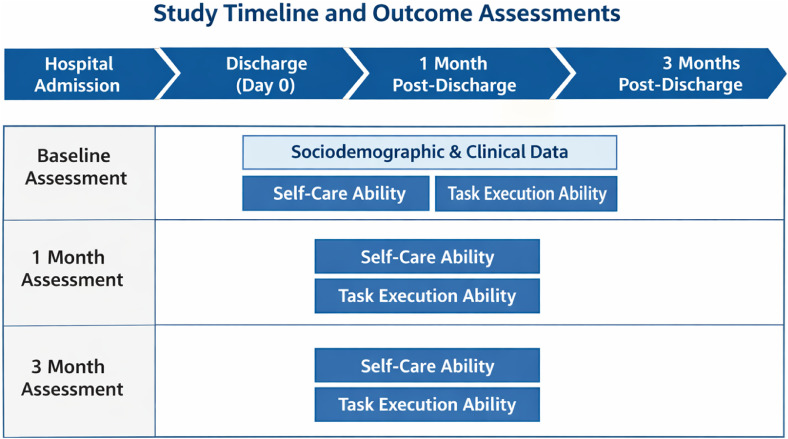
Study timeline and outcome assessentments.

Baseline sociodemographic and clinical characteristics were collected within 72 hours of hospital admission, including age, sex, living status, marital status, educational level, monthly income, heart function classification, and number of comorbidities. The present study was a secondary analysis of the parent randomized controlled trial and focused on short-term behavioral outcomes within the first 3 months after discharge. In this analysis, the behavioral outcomes of interest were self-care ability and task execution ability. Assessments were conducted at baseline, 1 month after discharge, and 3 months after discharge. The timeline of outcome assessments is illustrated in Fig. 2.

#### 2.4.1 Self-care ability

Self-care ability was measured using the Chinese version of the European Heart Failure Self-care Behavior Scale (EHFScBS-9), originally developed by Jaarsma et al. (Jaarsma et al., 2009) and subsequently translated and validated in Chinese populations (Yu et al., 2011). The scale consists of nine items assessing key aspects of heart failure self-care behaviors, with each item rated on a five-point Likert scale**. In the present analysis, item scores were reverse-coded and transformed so that higher total scores indicated better self-care ability, in order to maintain consistency with the direction of task execution ability and to facilitate interpretation of longitudinal changes.** Total scores range from 9 to 45. For descriptive and analytical purposes, the nine items were conceptually grouped into three dimensions: symptom monitoring and response, treatment adherence, and proactive health behavior and functional maintenance.

#### 2.4.2 Task execution ability

Task execution ability was assessed using a structured task performance scale designed to evaluate patients’ ability to independently perform essential self-management tasks for CHF. The instrument includes six representative tasks related to daily disease management, namely body weight monitoring, fluid intake measurement, recognition of prescribed diuretics, identification of high-salt foods, assessment of peripheral edema, and adjustment of diuretic dosage in response to symptom or weight changes. Each task was scored using a standardized scoring system, with higher scores indicating better task execution ability. The development procedures and psychometric properties of this instrument have been reported in previous studies (Zhu et al., 2025; Zhu et al., 2025). Guided by heart failure self-care theory and the symptom-oriented self-management process (Riegel et al., 2022), task execution ability was conceptually organized into three functional dimensions: symptom monitoring, symptom assessment, and symptom management and maintenance. Symptom monitoring included independent body weight measurement with accurate recording and precise measurement and documentation of daily fluid intake. Symptom assessment referred to the correct evaluation of peripheral edema based on observed physical changes. Symptom management and maintenance encompassed identification of prescribed diuretics, recognition of high-salt foods, and appropriate adjustment of diuretic dosage in response to monitored symptoms and weight changes. This conceptual categorization was used to facilitate interpretation of execution performance and its longitudinal changes, rather than to redefine the original scoring structure of the scale.

#### 2.4.3 Quality control

To ensure data quality and measurement consistency, both groups were managed in the same ward by the same nursing team, and all research nurses received standardized training before study initiation. Outcome assessments were performed by trained assessors who remained blinded to group allocation, and nurses responsible for intervention delivery were not involved in outcome assessment. Standardized administration procedures were applied at each time point, and questionnaires were checked on site for completeness and logical consistency. Data were entered independently by two researchers and cross-verified. Follow-up assessments were conducted according to a predefined schedule, and intervention fidelity was monitored regularly throughout the study period.

### 2.5 Statistical analysis

Continuous variables were assessed for normality and are presented as means with standard deviations (SD) or medians with interquartile ranges (IQR), as appropriate. Categorical variables are reported as frequencies and percentages. Baseline characteristics between groups were compared using independent-samples t tests or Mann–Whitney U tests for continuous variables and chi-square or Fisher’s exact tests for categorical variables. Longitudinal changes in self-care ability and task execution ability were analyzed using linear mixed-effects models, with group, time (baseline, 1 month, and 3 months), and their interaction included as fixed effects and a random intercept specified for each participant. The group × time interaction was used to examine differences in outcome trajectories between groups. Model estimates are reported as β coefficients with corresponding F statistics and two-sided P values. Standardized effect sizes (standardized β) were calculated to quantify the magnitude of intervention effects. To improve clinical interpretability, absolute changes from baseline to follow-up and between-group differences at each time point were also examined descriptively. Because baseline body weight differed between groups whereas body mass index did not, sensitivity analyses were conducted to examine the robustness of the main findings. Baseline body weight and baseline body mass index were entered separately as covariates in the mixed-effects models, and the group-by-time interaction terms were compared across models. For self-care ability, mean score changes were interpreted in relation to the possible score range of the transformed EHFScBS-9. For task execution ability, median changes were interpreted in relation to the number of independently performed self-management tasks. For non-normally distributed outcomes (task execution ability), descriptive statistics are presented as medians and interquartile ranges, and nonparametric tests were used for between-group comparisons at each time point. All analyses were performed using R software (version 2024.04.2+764), and a two-sided P value < 0.05 was considered statistically significant. In addition to overall scores, domain-level analyses were conducted for self-care ability and task execution ability. Each domain was analyzed using the same linear mixed-effects modeling framework with group, time, and group-by-time interaction terms and participant-level random intercepts. These analyses were predefined to characterize domain-specific intervention effects and did not alter the original scoring structure of the scales. Because domain analyses were considered supportive, no multiplicity adjustment was applied. All analyses were performed using R software (version 2024.04.2+764), and a two-sided P value < 0.05 was considered statistically significant.

## 3 Results

### 3.1 Baseline characteristics of participants

A total of 119 patients with CHF were included in the present analysis, comprising 65 in the control group and 54 in the intervention group. Baseline demographic and clinical characteristics are presented in Table 2. Most baseline demographic and clinical characteristics were comparable between groups. No statistically significant between-group differences were observed in age, sex, living status, marital status, educational level, monthly income, heart function classification, or number of comorbidities. However, body weight differed significantly between groups, while body mass index did not. Therefore, the baseline difference in absolute body weight was interpreted cautiously and further examined in sensitivity analyses.

**Table 2:** Baseline characteristics of participants in the two groups.

Variable	Total (n= 119)	Control group (n = 65)	Intervention group (n = 54)	*P* value
Age, years, mean (SD)	69.43 (11.84)	68.40 (12.81)	70.67 (10.54)	0.300
Intrinsic capacity total score, mean (SD)	97.43 (15.28)	96.43 (14.54)	98.63 (16.18)	0.437
Locomotion domain, mean (SD)	32.46 (6.87)	32.63 (6.72)	32.26 (7.10)	0.770
Vitality domain, mean (SD)	13.30 (2.37)	13.71 (2.23)	12.81 (2.46)	0.040
Sensory domain, mean (SD)	16.67 (2.67)	16.20 (2.70)	17.24 (2.54)	0.034
Cognition domain, mean (SD)	12.83 (5.07)	12.05 (5.12)	13.78 (4.90)	0.063
Psychological domain, mean (SD)	22.16 (4.22)	21.85 (3.98)	22.54 (4.50)	0.376
Grip strength–weight index (left), mean (SD)	26.73 (11.47)	27.05 (10.93)	26.35 (12.19)	0.741
Grip strength–weight index (right), mean (SD)	26.57 (13.39)	27.83 (13.51)	25.05 (13.21)	0.260
Height, cm, mean (SD)	162.61 (9.17)	164.04 (9.29)	160.90 (8.80)	0.063
Weight, kg, mean (SD)	66.19 (15.18)	68.89 (14.87)	62.95 (15.04)	0.033
Body mass index, kg/m², mean (SD)	24.78 (4.17)	25.38 (4.07)	24.06 (4.23)	0.088
Emergency visit, n (%)				1.000
No	107 (89.9)	58 (89.2)	49 (90.7)	
Yes	12 (10.1)	7 (10.8)	5 (9.3)	
Sex, n (%)				0.813
Male	73 (61.3)	41 (63.1)	32 (59.3)	
Female	46 (38.7)	24 (36.9)	22 (40.7)	
Living alone, n (%)				0.834
No	99 (83.2)	55 (84.6)	44 (81.5)	
Yes	20 (16.8)	10 (15.4)	10 (18.5)	
Marital status, n (%)				0.640
Never married	2 (1.7)	1 (1.5)	1 (1.9)	
Divorced	1 (0.8)	1 (1.5)	0 (0.0)	
Widowed	16 (13.4)	7 (10.8)	9 (16.7)	
Married	100 (84.0)	56 (86.2)	44 (81.5)	

Data are presented as mean (SD) for continuous variables and n (%) for categorical variables. Between-group comparisons were performed using independent-samples t tests for continuous variables and χ² tests or Fisher’s exact tests (when expected cell counts were <5) for categorical variables. A two-sided P value <0.05 was considered statistically significant.

### 3.2 Longitudinal trajectories of self-care ability and task execution ability

**Fig. 3:**
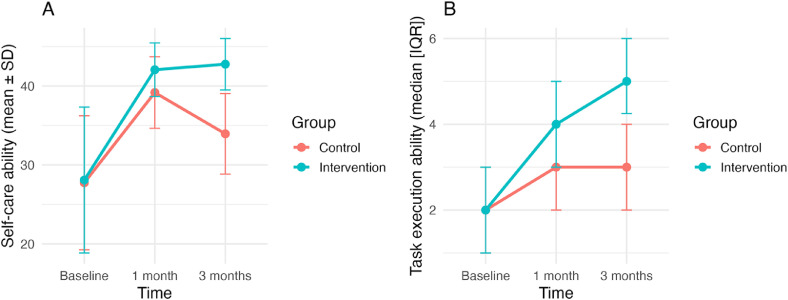
Longitudinal changes in self-care ability and task execution ability in the intervention and control groups.

Distinct longitudinal changes were observed in self-care ability and task execution ability during the 3-month follow-up period (Table 3; Fig. 3). For self-care ability, linear mixed-effects analysis showed significant main effects of group and time, as well as a significant interaction between group and time (Fgroup = 11.65, P < 0.001; Ftime = 5.74, P = 0.003; interaction P < 0.001). Both groups improved at 1 month after discharge; however, the intervention group maintained higher self-care scores at 3 months, whereas the control group showed a partial decline. The magnitude of this difference was clinically interpretable. From baseline to 3 months, the mean self-care ability score increased by 14.67 points in the intervention group and by 6.20 points in the control group, resulting in an approximate between-group difference of 8.82 points at 3 months. Considering the transformed EHFScBS-9 score range of 9–45, this difference represents a substantial separation in self-care performance during the early post-discharge period.

**Table 3:** Comparison of self-care ability and task execution ability between the two groups at different time points.

Group	Self-care ability (mean ± SD)			Task execution ability (median [IQR])		
	Baseline	1 month	3 months	Baseline	1 month	3 months
Control group (n=65)	27.74 ± 8.49	39.17 ± 4.53	33.94 ± 5.10	2.0 [1.0–3.0]	3.0 [2.0–3.0]	3.0 [2.0–4.0]
Intervention group (n=54)	28.09 ± 9.24	42.06 ± 3.40	42.76 ± 3.26	2.0 [1.0–3.0]	4.0 [3.0–5.0]	5.0 [4.25-6.0]
*t* ;Z / χ²	0.828	<0.001	<0.001	0.91	0.014	<0.001
*P* value	0.828	<0.001	<0.001	0.91	0.014	<0.001

Data are presented as mean ± standard deviation or median (interquartile range), as appropriate. Longitudinal changes were examined using repeated-measures analysis. For self-care ability, significant main effects of group (F = 11.65, P < 0.001) and time (F = 5.74, P = 0.003), as well as a significant group × time interaction (F = 8.10, P < 0.001), were observed. For task execution ability, no significant main effect of group was found (F = 0.01, P = 0.91); however, significant main effects of time (F = 36.19, P < 0.001) and a significant group × time interaction (F = 26.91, P < 0.001), were identified.

(A) Changes in self-care ability scores, presented as mean ± standard deviation, at baseline, 1 month, and 3 months after discharge. (B) Changes in task execution ability scores, presented as median [interquartile range], at the same time points. Control = usual care; Intervention = nurse-led age-friendly graded symptom-recognition intervention.

For task execution ability, no significant overall group main effect was observed (P = 0.91). However, significant time effects and a significant interaction between group and time were identified (Ftime = 36.19, P < 0.001; interaction P < 0.001). Task execution scores increased over time in both groups, but the improvement was more pronounced and sustained in the intervention group, resulting in progressive divergence between groups by 3 months. In practical terms, the intervention group improved from a median of 2.0 tasks at baseline to 5.0 tasks at 3 months, whereas the control group improved from 2.0 to 3.0 tasks. Thus, by 3 months, patients in the intervention group were able to perform approximately two additional self-management tasks compared with those receiving usual care. Given that the task performance scale includes six essential self-management tasks, this difference suggests a clinically meaningful improvement in patients’ operational capacity for daily heart failure self-management.

Sensitivity analyses adjusting for baseline body weight and baseline body mass index yielded consistent findings. The group-by-time interaction remained significant for both self-care ability and task execution ability after adjustment, suggesting that the observed longitudinal differences were robust to baseline anthropometric differences,as shown in Supplementary Table S1.

### 3.3 Longitudinal changes in the dimensions of self-care ability and task execution ability

Dimension-specific analyses showed heterogeneous patterns of change across domains of self-care ability (Table 4-1; Fig. 4-1). In the proactive health behavior and functional maintenance dimension, both groups improved at 1 month; however, scores declined at 3 months in the control group, whereas the intervention group maintained relatively stable high levels. In the treatment adherence dimension, both groups improved over time, but the intervention group showed greater stability and sustained gains at 3 months, while the control group showed mild fluctuation after the initial improvement. In the symptom monitoring and response dimension, the intervention group demonstrated a continuous upward trend across all time points, whereas the control group improved at 1 month but declined by 3 months, resulting in an increasing between-group difference over time.

**Table 4-1:** Comparison of self-care ability dimensions between groups at baseline, 1 month, and 3 months (Mean ± SD).

Dimensions	Group	baseline	1 month	3month
Proactive Health and Functional Maintenance	Control group	2.58 ± 1.30	4.38 ± 1.00	3.25 ± 1.15
	Intervention group	3.04 ± 1.35	4.65 ± 0.59	4.65 ± 0.73
Treatment Adherence	Control group	3.35 ± 0.94	4.43 ± 0.53	3.90 ± 0.62
	Intervention group	3.41 ± 1.00	4.69 ± 0.46	4.78 ± 0.39
Symptom Monitoring and Response	Control group	3.06 ± 1.13	4.35 ± 0.60	3.82 ± 0.62
	Intervention group	2.97 ± 1.19	4.67 ± 0.44	4.76 ± 0.38

Fig. 4-1 Longitudinal changes in self-care ability dimensions over time.Mean scores of proactive health & functional maintenance, treatment adherence, and symptom monitoring & response are shown at baseline, 1 month, and 3 months for the control and intervention groups. Data are presented as mean values. Group assignment was based on intervention status.

**Fig. 4-1:**
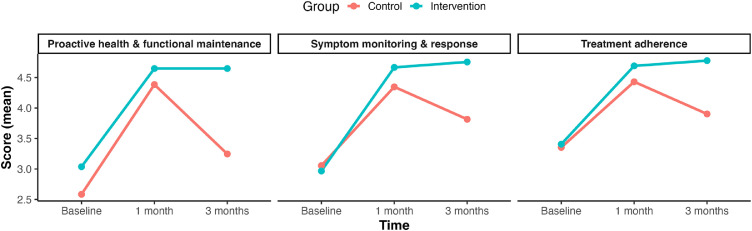
Longitudinal changes in the dimensions of self-care ability in the intervention and control groups.

Changes over time in the three dimensions of self-care ability, including proactive health and functional maintenance, symptom monitoring and response, and treatment adherence, at baseline, 1 month, and 3 months after discharge. Scores are presented as mean values. Control = usual care; Intervention = nurse-led age-friendly graded symptom-recognition intervention.

Distinct patterns were also observed across dimensions of task execution ability (Table 4-2; Fig. 4-2). The symptom assessment dimension showed high median scores in both groups from baseline onward, with minimal variation over time. In the symptom management and maintenance dimension, both groups improved by 1 month and maintained this level at 3 months, although variability was lower in the intervention group, suggesting greater stability of performance. In contrast, the symptom monitoring dimension showed the clearest divergence between groups. Although both groups improved at 1 month, the control group declined by 3 months, whereas the intervention group maintained maximal median scores, indicating better sustainability of monitoring behaviors. These dimension-level findings further support the clinical relevance of the overall effects. The most consistent between-group separation was observed in symptom monitoring-related domains, suggesting that the intervention primarily strengthened behaviors requiring repeated observation, recording, and action initiation rather than producing uniform improvement across all self-management components.

**Table 4-2:** Comparison of task execution ability dimensions between groups at baseline, 1 month, and 3 months (Median [IQR]).

Dimension	Group	Baseline	1 Month	3 Months
Symptom Management and Maintenance	Control group	0.33 [0.00, 0.33]	0.67 [0.33, 0.67]	0.67 [0.33, 0.67]
	Intervention group	0.33 [0.00, 0.67]	0.67 [0.67, 1.00]	0.67 [0.67, 1.00]
Symptom Monitoring	Control group	0.50 [0.00, 0.50]	1.00 [0.50, 1.00]	0.50 [0.00, 0.50]
	Intervention group	0.00 [0.00, 0.50]	1.00 [0.50, 1.00]	1.00 [0.50, 1.00]
Symptom Assessment	Control group	1.00 [1.00, 1.00]	1.00 [0.00, 1.00]	1.00 [1.00, 1.00]
	Intervention group	1.00 [1.00, 1.00]	1.00 [1.00, 1.00]	1.00 [1.00, 1.00]

Fig. 4-2 Trends in task execution ability dimensions over time.Median scores for symptom monitoring, symptom management & maintenance, and symptom assessment are shown at baseline, 1 month, and 3 months for the control and intervention groups. Circles indicate the control group and triangles indicate the intervention group. Overlapping markers reflect identical median values at certain time points.

**Fig. 4-2:**
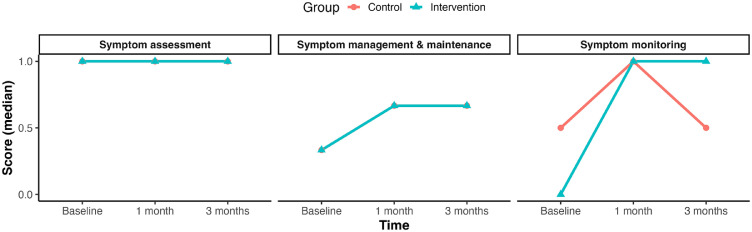
Longitudinal changes in the dimensions of task execution ability in the intervention and control groups.

Changes over time in the three dimensions of task execution ability, including symptom assessment, symptom management and maintenance, and symptom monitoring, at baseline, 1 month, and 3 months after discharge. Scores are presented as median values. Control = usual care; Intervention = nurse-led age-friendly graded symptom-recognition intervention.

## 4 Discussion

This study examined the behavioral effects of a nurse-led age-friendly graded symptom-recognition intervention in older patients with chronic heart failure during the vulnerable post-discharge transition period. Three main findings emerged. First, both self-care ability and task execution ability showed significantly different longitudinal trajectories between groups, indicating that the intervention influenced not only outcome levels but also patterns of change over time. Second, the intervention group demonstrated more sustained improvement, whereas the control group showed partial decline by 3 months, suggesting that routine post-discharge education alone may be insufficient to maintain behavioral gains. Third, the effects of the intervention were not uniform across dimensions. In both scales, domains related to symptom monitoring and action-oriented management appeared more responsive than those involving more stable or less behaviorally demanding functions. Taken together, these findings suggest that the intervention may have exerted its effects by strengthening the connection between symptom recognition, response selection, and daily enactment of self-management behaviors. Beyond statistical significance, the observed effects appear to have practical relevance for nursing care and post-discharge self-management. The approximately 8.82-point between-group difference in transformed self-care ability at 3 months suggests that the intervention helped patients sustain broader self-care behaviors after discharge, rather than showing the decline observed in the control group. More importantly, the two-task median difference in task execution ability indicates that patients receiving the intervention could independently perform more concrete disease-management tasks, such as symptom monitoring, recording, edema assessment, or medication-related actions. This distinction is clinically meaningful because previous evidence in older patients with heart failure has shown that self-reported self-care and actual task performance are only weakly correlated, and that poorer task performance is associated with adverse outcomes (Vidán et al., 2019). Therefore, the improvement in task execution ability observed in the present study should be interpreted not simply as a numerical change, but as an increase in patients’ operational capacity to translate symptom cues and care instructions into daily self-management actions. At the same time, the clinical interpretation of these effect sizes should remain cautious. Established minimal clinically important differences are not available for the transformed self-care score or for the structured task execution scale used in this study. In addition, the study was conducted in a single center and focused on older patients with chronic heart failure during the early post-discharge transition period. Therefore, although the magnitude and consistency of the observed effects support clinical relevance, the generalizability of these findings should be confirmed in multicenter studies with longer follow-up and direct assessment of downstream clinical outcomes.

For self-care ability, the significant main effects of group and time, together with the significant interaction between group and time, indicate that the intervention altered the overall developmental trajectory of self-care after discharge. Although both groups improved at 1 month, only the intervention group maintained or further improved performance at 3 months, whereas the control group showed partial regression. This pattern suggests that self-care behavior in older patients with CHF may be temporarily enhanced after hospitalization but is difficult to sustain without continued behavioral reinforcement. This interpretation is consistent with the nature of heart failure self-care, which depends on repeated cycles of symptom perception, interpretation, and action rather than on one-time acquisition of knowledge alone (Auld et al., 2018). In the present study, the age-friendly graded symptom-recognition framework may have facilitated this process by reducing ambiguity in symptom appraisal and by linking symptom changes with specific behavioral responses. This mechanism is especially relevant in older patients, who often have difficulty converting generalized advice into concrete action under conditions of symptom uncertainty. The dimension-specific findings provide further insight into how the intervention improved self-care ability. In the proactive health behavior and functional maintenance dimension, both groups improved at 1 month, but only the intervention group maintained this gain at 3 months, whereas the control group showed a clear decline. This suggests that behaviors requiring sustained initiative and continuous incorporation into daily routines may initially respond to discharge education but are unlikely to remain stable without structured support. A similar pattern was observed in the treatment adherence dimension. Although both groups improved over time, the intervention group demonstrated greater stability, whereas the control group showed mild regression after the initial post-discharge gain. This finding implies that treatment adherence in older patients with CHF is shaped not only by knowledge of medication and diet but also by the ability to continuously relate symptom changes to treatment-related decisions. The most pronounced divergence was observed in the symptom monitoring and response dimension, in which the intervention group showed a continuous upward trajectory while the control group improved early and then declined by 3 months. Because symptom monitoring and response lie at the core of heart failure self-care, this result suggests that the intervention most effectively strengthened those components of self-care that require repeated vigilance, situational judgment, and active engagement. In this sense, the graded symptom-recognition approach appears to have acted less as a general educational strategy and more as a practical behavioral support system for maintaining symptom-related self-management.

Task execution ability showed a related but distinct longitudinal pattern. Although no significant overall group main effect was observed, the pronounced time effect and significant interaction between group and time indicate that execution capacity evolved differently across groups and diverged earlier than self-care ability. This finding is clinically important because task execution ability reflects whether patients can translate symptom cues and care instructions into concrete behaviors, including monitoring, documenting, identifying relevant disease-management information, and initiating appropriate responses (Kurtz et al., 2025). In older patients with CHF, this ability is often impaired by deficits in executive function, working memory, and action planning, even when general disease knowledge is present (Fan et al., 2023). Prior studies have similarly shown that poor self-management in heart failure is more strongly associated with deficits in behavioral enactment than with insufficient knowledge alone (Vidán et al., 2019; Jacobson et al., 2018). The present findings extend this evidence by showing that task execution ability is modifiable over time and is particularly responsive to repeated, structured, nurse-led support. The earlier divergence observed in execution trajectories also suggests that this ability may be more sensitive than self-care self-report measures to early changes in behavioral performance. Several aspects of the intervention may explain why task execution ability improved earlier and more clearly than self-care ability. First, the intervention involved repeated one-to-one demonstration, supervised practice, and staged reinforcement during hospitalization, all of which may have promoted procedural learning rather than simple declarative understanding (Gebauer et al., 2023). Second, follow-up intensity after discharge was adjusted according to adherence and accuracy, which likely prevented early performance decay and supported progressive skill stabilization. Third, the graded symptom-recognition structure linked symptom levels to predefined action strategies, thereby reducing decisional uncertainty and lowering the threshold for behavioral initiation. This is particularly important in older patients with CHF, who often notice symptom changes but remain uncertain about their severity, significance, and the appropriate next step, which may contribute to delayed healthcare seeking and inadequate response (Cheng et al., 2020). From the perspective of task representation, the intervention may have improved performance by transforming symptom-related self-management from an ill-defined and cognitively demanding problem into a more structured and externally supported task. By explicitly mapping salient symptom cues onto corresponding response options, the graded tool simplified how patients mentally represented the task, making strategy selection clearer and more actionable. This interpretation is consistent with evidence that task representation influences strategy generation, selection, and problem-solving performance, particularly under conditions requiring monitoring and repeated decision-making (Xie and Moss, 2025) . In older patients with CHF, whose symptom management is often constrained by reduced attentional control, working memory, and confidence in symptom appraisal, such representational simplification may be especially valuable. The intervention therefore appears to have functioned not only as an educational program but also as a behavioral scaffold that facilitated the translation of symptom information into executable action. The dimension-level patterns of task execution ability further clarify this mechanism. Among the three dimensions, symptom monitoring showed the clearest divergence over time. Both groups improved at 1 month, but only the intervention group maintained this improvement at 3 months, whereas the control group declined. This suggests that symptom monitoring is a highly practice-dependent behavior that requires repetition, procedural consistency, and sustained attention, making it particularly responsive to structured reinforcement. By contrast, the symptom management and maintenance dimension improved in both groups and remained relatively stable, but the intervention group showed greater consistency of performance, indicating that structured support may improve not only whether these tasks are performed but also how reliably they are carried out. The symptom assessment dimension changed little across time in either group, with consistently high median values, suggesting limited room for improvement and a likely ceiling effect. This pattern indicates that not all aspects of execution are equally modifiable. Compared with symptom assessment, which may already have been relatively preserved in this sample, domains involving repeated action and behavioral enactment appear more trainable through nurse-led intervention. This distinction is clinically meaningful because it suggests that future programs may achieve greater benefit by prioritizing trainable execution behaviors rather than assuming that all self-management domains respond equally to education and follow-up.

The present findings are broadly consistent with prior studies suggesting that structured support for symptom recognition and response can improve heart failure-related self-management behaviors. This may also be interpreted in light of cognitive task implementation research showing that explicitly instructed rules are executed more efficiently than rules that must be inferred, particularly during the early phase of task performance. In the present study, the graded symptom-recognition tool made response rules explicit by directly linking symptom levels to corresponding actions, thereby reducing the cognitive demands of inference and facilitating more immediate and reliable behavioral implementation (Zhu et al., 2026; Pereg and Meiran, 2021). At the same time, this study extends the existing literature in several important ways. By incorporating both self-care ability and task execution ability, it captured not only patients’ self-reported behavioral capacity but also their more proximal ability to perform concrete disease-management tasks. In addition, the use of repeated measurements and mixed-effects modeling enabled characterization of behavioral trajectories rather than simple baseline-to-endpoint differences. These strengths are particularly relevant in older patients with CHF, whose barriers to self-management often include symptom ambiguity, reduced confidence in symptom interpretation, and increased cognitive burden (Wang et al., 2025; Prell et al., 2023). In this context, the intervention may be understood as restructuring the symptom-perception-action pathway by making self-management decisions more concrete, less cognitively demanding, and more reproducible, a mechanism consistent with evidence that visual and simplified support tools can improve understanding and behavior in individuals with limited processing capacity or low health literacy (Mbanda et al., 2021). Collectively, these findings suggest that age-friendly graded symptom-recognition strategies may represent a valuable component of transitional care, particularly during the vulnerable post-discharge transition period when behavioral divergence begins to emerge and reinforcement may be most influential.

This study should be interpreted in light of several limitations. First, this was a single-center study with a relatively short follow-up period; therefore, the generalizability and durability of the observed behavioral effects require confirmation in larger multicenter studies with longer follow-up. Second, although randomization was used, baseline body weight differed between groups. However, body mass index did not differ significantly, and absolute body weight alone should not be interpreted as direct evidence of greater clinical decompensation because it may reflect body size, nutritional status, obesity, muscle mass, or fluid retention. Sensitivity analyses adjusting separately for baseline body weight and baseline body mass index yielded consistent findings, suggesting that the observed longitudinal effects were unlikely to be driven by baseline anthropometric imbalance. Nevertheless, future studies with more detailed indicators of congestion, body composition, and nutritional status are needed to clarify whether baseline physical status modifies intervention effects. Third, although the observed findings suggest plausible behavioral mechanisms, unmeasured factors such as caregiver involvement, cognitive heterogeneity, health literacy, and digital engagement may also have influenced self-care and task execution trajectories. Fourth, self-care measures are inherently sensitive to response tendencies and scoring direction, although the scoring direction was clarified and consistently applied in the present analysis. Finally, the present secondary analysis focused on behavioral outcomes and did not directly test whether improvements in task execution ability mediated downstream clinical outcomes. Future studies should examine whether improved task execution ability translates into reduced delayed healthcare seeking, rehospitalization, or symptom-related service utilization, and should determine which execution domains are most responsive to structured nurse-led intervention.

## 5 Conclusion

Among older patients with chronic heart failure, a nurse-led age-friendly graded symptom-recognition intervention was associated with improved longitudinal patterns of both self-care ability and task execution ability during the first 3 months after discharge. The observed interaction between group and time suggests that the intervention influenced not only outcome levels but also their evolution over time, with progressive gains in self-care ability and earlier divergence in task execution ability. These findings highlight the value of integrating structured symptom-recognition and task-oriented support into transitional care. Task execution ability may represent a clinically meaningful and modifiable behavioral target in heart failure self-management. Further studies with longer follow-up are needed to determine whether these behavioral improvements translate into downstream clinical benefits, including reduced rehospitalization.

## Study registration details

This study was registered with the Chinese Clinical Trial Registry (ChiCTR) (Registration No. ChiCTR2500106241). Registration date: 21 July 2025.

## Ethics approval and consent to participate

The authors are accountable for all aspects of the work in ensuring that questions related to the accuracy or integrity of any part of the work are appropriately investigated and resolved. This study was conducted in accordance with the Declaration of Helsinki (as revised in 2013), and the study protocol was approved by the ethics committee of *** hospital, *** university school of medicine (Approval No. 2025-Y0513). Informed consent was obtained from all participants before survey initiation.

## Funding statements

1) The Health Commission of Zhejiang Province Project in China (No.2024KY1100)
2) The nursing research project of Sir Run Run Shaw hospital, ZheJiang university school of medicine (2024HLKY01)
3) Zhejiang University “Double First-Class” Construction Special Project for Advantageous and Distinctive Disciplines (Grant No. HL2024011)

## CRediT authorship contribution statement

**Haixiang Zhu:** Writing – review & editing, Writing – original draft, Methodology, Funding acquisition, Conceptualization. **Yan Li:** Data curation. **Xiaoxue Han:** Investigation, Data curation. **Jinxuan Chen:** Investigation, Data curation. **Ruiting Wang:** Writing – original draft. **Xiuruo Zhu:** Visualization, Conceptualization.

## Declaration of competing interest

The authors declare that they have no known competing financial interests or personal relationships that could have appeared to influence the work reported in this paper. All authors have reviewed the manuscript and consent to its publication. The authors have no conflicts of interest to declare.

## Data Availability

All data supporting the findings of this study are included in the article. No additional data are available.

## Appendix Supplementary materials

Image, application 1

Image, application 2
