# Supplementary material for: Trajectories of self-care and task execution abilities among older patients with chronic heart failure following a nurse-led intervention: A longitudinal mixed-effects analysis
Source: Int J Nurs Stud Adv. 2026 Jul 14;11:100619. doi: 10.1016/j.ijnsa.2026.100619 (PMC13393736; doi:10.1016/j.ijnsa.2026.100619)
Supplement: Supplementary file 1 [file mmc1.docx]

**Supplementary File 1. Age-friendly graded symptom-recognition tool for patients with chronic heart failure.**


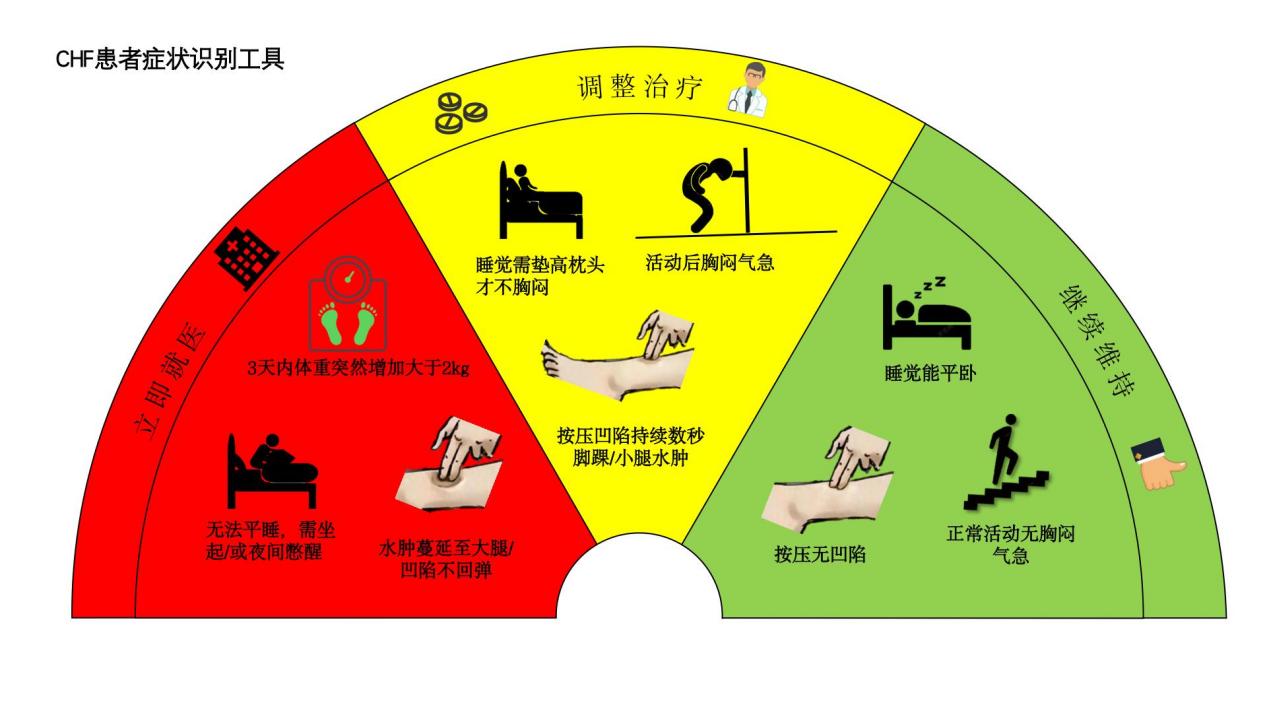

This age-friendly graded symptom-recognition tool classifies common symptoms of chronic heart failure into three levels according to severity and corresponding response recommendations. The green zone indicates relatively stable status and suggests continuation of routine self-care and monitoring. The yellow zone indicates possible symptom worsening and suggests adjustment of treatment or prompt contact with healthcare professionals. The red zone indicates severe or rapidly worsening symptoms and suggests immediate medical attention. The tool incorporates common symptom indicators, including orthopnea, exertional chest tightness or dyspnea, rapid weight gain within 3 days, and the severity of lower-limb oedema, to support symptom recognition and response decision-making in older patients with chronic heart failure.

**Instructions for use of the age-friendly graded symptom-recognition tool**

This tool is intended to support older patients with chronic heart failure in identifying symptom severity and selecting an appropriate response. Patients or caregivers are asked to review the symptoms shown in the figure and determine which color zone best matches the patient’s current condition.

**Green zone (continue self-care and monitoring):**
If the patient is able to lie flat while sleeping, has no chest tightness or shortness of breath during normal activities, and has no pitting oedema on pressure, the condition is considered relatively stable. In this situation, the patient should continue routine self-care, including daily monitoring and prescribed treatment.

**Yellow zone (adjust treatment / seek timely advice):**
If the patient needs a higher pillow to sleep without chest tightness, develops chest tightness or shortness of breath after activity, or shows ankle or lower-leg oedema with pitting that persists for several seconds, the condition may be worsening. In this situation, the patient should increase attention to symptom monitoring, consider treatment adjustment according to professional guidance, and contact healthcare professionals when necessary.

**Red zone (seek immediate medical attention):**
If the patient is unable to lie flat and must sit up to breathe or wakes at night due to breathlessness, experiences a sudden body weight gain of more than 2 kg within 3 days, or develops oedema extending to the thigh with non-rebounding pitting, urgent medical attention is required.

The tool should be used together with routine self-monitoring practices, including symptom observation, body weight recording, and communication with healthcare providers. It is intended as an age-friendly decision-support aid rather than a substitute for professional clinical judgment.
