# Supplementary material for: Trajectories of self-care and task execution abilities among older patients with chronic heart failure following a nurse-led intervention: A longitudinal mixed-effects analysis
Source: Int J Nurs Stud Adv. 2026 Jul 14;11:100619. doi: 10.1016/j.ijnsa.2026.100619 (PMC13393736; doi:10.1016/j.ijnsa.2026.100619)
Supplement: Supplementary file 2 [file mmc2.docx]

**Supplementary Table S1 Sensitivity analyses adjusted for baseline body weight and body mass index**

| Outcome | Model | F value | P value |
| --- | --- | --- | --- |
| Self-care ability | Adjusted for baseline body weight | 15.13 | <0.001 |
| Self-care ability | Adjusted for baseline body mass index | 15.13 | <0.001 |
| Task execution ability | Adjusted for baseline body weight | 13.33 | <0.001 |
| Task execution ability | Adjusted for baseline body mass index | 13.34 | <0.001 |

Note. Sensitivity analyses were conducted using mixed-effects models. Baseline body weight and baseline body mass index were entered separately as covariates in the adjusted models. The F value and P value refer to the group-by-time interaction term. BMI, body mass index.
